# Hematogenous infantile infection presenting as osteomyelitis and septic arthritis: a case report

**DOI:** 10.4076/1757-1626-2-8293

**Published:** 2009-07-21

**Authors:** Savas P Deftereos, Eleftheria Michailidou, Georgios K Karagiannakis, Stella Grigoriadi, Panos Prassopoulos

**Affiliations:** Department of Radiology, University Hospital of AlexandroupolisDemocritus University of Thrace, Alexandroupolis (Dragana), 68100Greece

## Abstract

The case of a 6-month old male infant presenting at the emergency department with fever and swelling at the left knee joint is discussed. Laboratory tests showed an inflammatory condition. Left knee plain radiograph demonstrated local soft tissue oedema. Percutaneous needle aspiration of articular fluid showed a positive culture for *Staphylococcus aureus*. The diagnosis of septic arthritis was confirmed. Because of inadequate response to treatment an MRI study was followed to evaluate possible abscesses. The presence of an abscess in the suprapatellar bursa was confirmed and an additional inflammatory process of the bone marrow was revealed, consistent with osteomyelitis. The pathophysiology, the imaging findings, the patient’s management and a review of septic arthritis and osteomyelitis coexistence are presented in this paper.

## Case presentation

**Figure 1.**
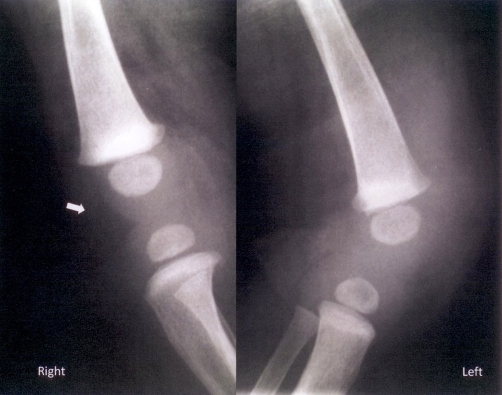
Plain radiograph in lateral projection of both knee joints. Indistinctness of soft tissue-fat iterface at the left knee joint is demonstrated suggesting presence of oedema. Note the normal right side with clear fat plane (arrow) imaging. There is also a radiolucent area at the left tibial epiphysis suggesting osteomyelitis.

**Figure 2.**
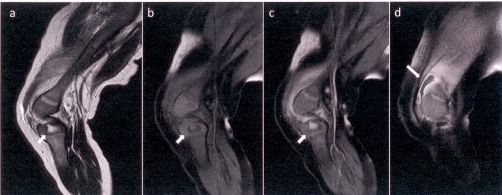
MRI study (sagittal projections). **(a)** T2-Weighted Images (WI). On the epiphysis of left tibia an oval area (arrow) of high signal intensity is demonstrated. **(b)** T1-WI with Fat Saturation demonstrates the same area (arrow) with intermediate to low signal intensity. **(c)** T1-WI with Fat Saturation (FS) after intravenous administration (IV) of paramagnetic substance. The lesion (arrow) avidly enhances. There is also soft tissue enhancement around the distal part of femur. **(d)** T1-WI with FS after IV of paramagnetic substance. Fluid collection with peripheral enhancement (arrow) in the suprapatellar pouch represents an abscess.

**Figure 3.**
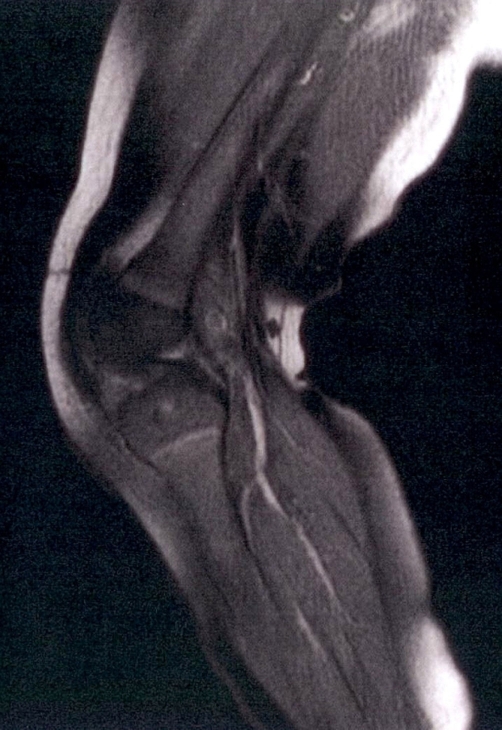
Follow up MRI (T1-WI, FS and IV in sagittal projection) with remarkable improvement of imaging findings.

A 6-month old Greek male infant presented at the emergency department. Swelling in the left knee joint persisting for the last five days was referred by the parents. There was no penetrating wound, fracture or other bony injury in child’s history. The infant had a temperature of 38°C (48 hours) before admission in the hospital. During the physical examination a warm, erythematous and swollen left knee was observed. Laboratory tests showed elevated white blood cell count (WBC), erythrocyte sedimentation rate (ESR) and C-reactive protein (CRP). Comparative plain radiograph of both knees (Figure 1) demonstrated local soft tissue oedema at left knee joint. A percutaneous needle aspiration of left knee joint fluid was performed giving a purulent fluid. Blood and joint fluid cultures were positive for *Staphylococcus aureus*. Because systematic symptoms were still there, even with lower but significant severity, after intravenous antibiotic therapy, an MRI study was performed. On the epiphysis of left tibia an oval area of high signal intensity on T2-weighted images (Figure 2a) and of intermediate to low signal intensity on T1-weighted images (Figure 2b) was revealed which avidly enhanced after intravenous administration of paramagnetic substance (Figure 2c). Ipsilateral soft tissue oedema was also demonstrated. A small fluid collection presenting peripheral enhancement after administration of paramagnetic substance was recognized in the suprapatellar pouch, a finding consistent with an abscess (Figure 2d). A splint was used to immobilize the left knee joint and modified intravenous antibiotic treatment was administered. Two days later the infant was feverless and in good general condition. The above findings were attributed to osteomyelitis with abscess formation. The patient was under conservative treatment for approximately a month. Follow-up MRI revealed remarkable improvement of imaging findings (Figure 3).

## Discussion

Osteomyelitis occurs more often in 18-24 month old children with male preponderance. *Staphylococcus aureus* is the most common causative agent in children and adults while streptococcus is most commonly found in infants and neonates. In 70% of cases the femoral bone and tibia are affected [1].

Microorganisms in most cases enter bone by the hematogenous route but direct introduction from a contiguous focus of infection, or penetrating wound could be causes of entrance. In infancy osteomyelitis and septic arthritis commonly occur together because, while in the older child the blood supply to the epiphysis and metaphysis is separate, in the neonate and young infant it is contiguous [2].

Hematogenous osteomyelitis in children affects mainly the rapidly growing and highly vascular metaphysis of long bones. Metaphyseal terminal arterial branches progress into a network of capillary loops and large venous sinusoids where blood flow is sluggish and functioning phagocytes are scarce [3]. These are optimal conditions for bacteria to inoculate. Bacterial septic emboli spread into the vascular channels, raising intraosseous pressure and obstructing the flow of blood [4]. Consequent ischemic necrosis of bone results in the formation of subperiosteal and soft tissue abscesses. Infection spreading occurs in joints where the articular capsule is attached to the periosteum beyond the edge of the articular cartilage. In those joints (shoulder, elbow, hip, knee joint) rupture of an abscess located in the metaphysis can lead to septic arthritis [5].

The presence of vascular connections between the metaphysis and the epiphysis makes infants particularly prone to septic arthritis of the adjacent joint [6]. Communicating vessels (transphyseal vessels) between the epiphysis and metaphysis transgress the growth plate (physis) and thus it is not uncommon for infection to extend from its primary site in the metaphysic to the epiphysis and then out into the joint space [6].

On plain films osteomyelitic lesions are not seen before 10 to 14 days after joint infection. Early finding and maybe the only findings are local soft tissue oedema and/or joint effusion with less than 5% of plain films revealing abnormal findings on presentation, less than 33% being positive at 1 week and 90% positive by 3-4 weeks. Late imaging findings on plain radiographs, especially in 3-4 week period, are bony destruction and following periosteal new bone deposition [7].

MRI is particularly suited for soft tissue for evaluation of early bone and soft tissue infection. At MRI examination osteomyelitis produces high signal intensity on T2-weighted images and low or intermediate signal intensity on T1-weighted images, avidly enhancing after intravenous paramagnetic substance administration. High signal on T2-weighted images represents replacement of marrow fat by fluid secondary to local exudates and oedema. Short Time Inversion Recovery (STIR) sequences are sensitive in demonstrating bone marrow lesions (alterations). STIR sequence results in improved contrast between marrow and infection. Generally anatomic detail provided by MRI is superior to radionuclide scans [8].

Based on the above we can conclude that in osteomyelitis deep oedema is detectable from the onset and bone changes are later presented. It is during this time that, if confirmation of osteomyelitis is required (negative joint fluid culture or inadequate response to therapy) the MRI study is useful. As noted above osteomyelitis and septic arthritis frequently coexist in infants and children. Most often septic arthritis is first suspected on the basis of fluid presence in a joint on plain films. Ultrasonography also can be used for the demonstration of joint fluid. It is important to diagnose septic arthritis quickly because intra-articular pus accumulation can lead to joint distention and compromise of blood supply. In addition, articular cartillage destruction occurs early and thus accurate diagnosis with subsequent drainage is of the essence. MRI plays a major role to demonstrate the extent of osteomyelitis and/or septic arthritis lesions and in the follow up [9].

## Abbreviations

CRP: C-reactive protein
ESR: erythrocyte sedimentation rate
MRI: Magnetic Resonance Imaging
STIR: Short Time Inversion Recovery
WBC: white blood cell count
